# White Meat Consumption, All-Cause Mortality, and Cardiovascular Events: A Meta-Analysis of Prospective Cohort Studies

**DOI:** 10.3390/nu13020676

**Published:** 2021-02-20

**Authors:** Roberta Lupoli, Marilena Vitale, Ilaria Calabrese, Annalisa Giosuè, Gabriele Riccardi, Olga Vaccaro

**Affiliations:** 1 Department of Clinical Medicine and Surgery, “Federico II” University, 80131 Naples, Italy; roberta.lupoli@unina.it (R.L.); marilena.vitale@unina.it (M.V.); ilariacalabrese@live.it (I.C.); annalisa.giosue@gmail.com (A.G.); 2 Department of Pharmacy, “Federico II” University, 80131 Naples, Italy; ovaccaro@unina.it

**Keywords:** meta-analysis, cohort studies, white meat, poultry, all-cause mortality, cardiovascular disease, coronary heart disease, stroke

## Abstract

The association of meat consumption with mortality and morbidity for non-communicable diseases has been extensively studied. However, the relation of white meat consumption with health outcomes remains controversial. The present meta-analysis was conducted to comprehensively analyze the available evidence on the consistency and strength of the association between the consumption of white meat, death from any cause and incidence of fatal and non-fatal cardiovascular (CV) events. PubMed, Web of Science, Scopus and Embase databases were searched for articles published up to April 30, 2020. We included prospective cohort studies reporting relative risks and pertinent 95% confidence intervals (CI) for all-cause mortality and/or CV events (fatal or non-fatal). A total of 22 studies were included in the meta-analysis. Eleven studies (14 data sets) reported data on all-cause mortality, 10 studies (15 datasets) on cardiovascular disease (CVD) mortality and 10 studies (11 datasets) on non-fatal CV events. When comparing the highest versus the lowest consumption of white meat, the pooled OR and pertinent 95% CI were 0.94 (0.90, 0.97, *p* < 0.001) for all-cause mortality, 0.95 (0.89, 1.01, *p* = 0.13) for CV mortality, and 0.99 (0.95, 1.02, *p* = 0.48) for non-fatal CV events. In conclusion, the study shows for the first time a robust and inverse association between white meat consumption and all-cause mortality and a neutral association with CV mortality and morbidity. This highlights the importance of differentiating the meat types for what concerns their health effects and suggests that white meat might be a healthier alternative to read and processed meat consumption.

## 1. Introduction

The relationship between dietary habits and chronic non-communicable diseases (NCDs) has been extensively investigated [1,2,3,4,5,6]. Although randomized trials with hard end points have not been feasible for most dietary factors, other lines of evidence, including long-term prospective observational studies and short-term trials with intermediate outcomes, have provided supporting evidence for potential causal relationships between dietary factors and health. A high-quality diet, comprising whole grains, fruits, vegetables, nuts, non-tropical vegetable oils, and fish, is one of the most important factors in preventing early death and non-communicable diseases worldwide [1,7], more than any other risk factors globally, including tobacco smoking [1,8,9]. On the contrary, high consumption of animal food sources (meat, in particular processed meat) and sodium intake are the main dietary risk factors for early death and disabilities due to NCDs [1]. A large body of evidence from prospective cohort studies has shown that high versus low meat consumption, is associated with an excess risk of all-cause and cause-specific mortality [10,11]. However, when type of meat consumed is analyzed disjointedly, different associations have been observed. In fact, while several reviews and meta-analyses have convincingly shown a positive association between red and processed meat consumption and risk of all-cause mortality [7,12,13,14,15], incidence of CVD, diabetes and some types of cancers [16,17,18,19,20,21], the association of white meat with mortality and morbidity for NCD is not clearly established [11,15,22,23]. There is evidence that the substitution of one daily serving of red meat with white meat, mainly poultry, is associated with a 19% reduction of cardiovascular risk [24], but this finding was not confirmed by other studies [11,15,22]. The most comprehensive work on this topic is a meta-analysis of prospective cohort studies on the relationship between white meat consumption and total mortality showing a weak inverse association in women when comparing the highest vs. the lowest consumption category [15]. In this same study, no association was reported with CVD mortality in either men or women and no data were provided on non-fatal CV events. As discussed by the authors, the small number, and the high heterogeneity of the meta-analyzed studies, does not allow to reach conclusive results. Following this work completed in 2014, several large prospective cohort studies have investigated the relationship of white meat consumption with health outcomes, some of these studies report also data on non-fatal CV endpoints [5,25,26,27,28,29,30,31,32]. We have carried out the present meta-analysis to comprehensively analyze the most updated available evidence on the consistency and strength of the association between the consumption of white meat, death from any cause and incidence of fatal or non-fatal cardiovascular events.

## 2. Materials and Methods

### 2.1. Search Strategy and Study Selection

A systematic research of all the prospective cohort studies published until April 30, 2020 on the association of white meat consumption with all-cause mortality, incidence of cardiovascular disease and coronary heart disease, was performed according to PRISMA (Preferred Reporting Items for Systematic reviews and Meta-Analyses) guidelines [25].

The research was carried out in the electronic databases (PubMed, Web of Science, Scopus, Embase) using the following key words in all possible combinations: (“poultry” or “white meat” or “chicken” or “turkey” or “rabbit”) and (“myocardial” or “coronary” or “mortality” or “cardiovascular” or “ischemic” or “stroke” or “cerebrovascular” or “death” or “fatal” or “fatality” or “events”) and (“meat” or “consumption” or “intake” or “serving”). The search was limited to human studies and had no language restrictions.

We included studies defining “white meat” as poultry (chicken, turkey, duck and goose) and rabbit. Exclusion criteria were: (i) retrospective studies; (ii) studies conducted on vegetarian people; and (iii) studies in which it was not possible to evaluate white meat alone because part of combined meals. When several publications of the same study were identified, only the most recent, or most detailed publication was used.

The reference lists of selected studies and reviews were also searched to identify additional articles not previously included. In addition, the lists of the retrieved articles were manually reviewed. In case of missing data, the Authors were contacted by e-mail to acquire the original data. This meta-analysis has been registered on Prospero (https://www.crd.york.ac.uk/PROSPERO (accessed on 26 January 2021)) with the registration number: CRD42020198126.

### 2.2. Data Extraction and Study Quality

Two reviewers (I.C. and A.G.) assessed the titles and abstracts of all identified studies and independently reviewed and extracted relevant data from each study, including first author and year of publication, country, study design, sample size, participant characteristics, follow-up duration, number of subjects in comparison groups, type of white meat, and main results of the outcomes investigated. In case of disagreement, a third investigator was consulted (M.V.). Discrepancies were resolved by consensus.

The evaluation of methodological quality of each study was performed with the Newcastle-Ottawa scale (NOS), which is specifically developed to assess quality of non-randomized observational studies [33]. The scoring system encompasses three major domains (selection, comparability, exposure) and a resulting score range between 0 and 8, a higher score representing a better methodological quality. Results of the NOS quality assessment are reported in Supplemental Table S1.

### 2.3. Statistical Analysis

The primary outcome was all-cause mortality defined as death for any cause (vascular and non-vascular). Secondary outcomes were CV mortality and non-fatal CV events (including ischemic heart disease, ischemic stroke, and hemorrhagic stroke). Data synthesis and statistical analysis were carried out using comprehensive meta-analysis (Version 3, Biostat, Englewood, NJ, USA, 2006) software. The pooled probability of all-cause mortality, cardiovascular mortality and cardiovascular events in subjects in the highest versus the lowest category of white meat consumption was expressed as OR with pertinent 95% confidence intervals (CI). The overall effect was tested by Z-scores, with *p* < 0.05 being considered statically significant.

Statistical heterogeneity among studies was assessed by chi square Cochran’s Q test and with I^2^ statistic, which measure the inconsistency across study results and describe the proportion of total variation in study estimates due to heterogeneity rather than sampling error. In detail, an I^2^ value of 25% corresponds to low, 25–50% to moderate, and 50% to high heterogeneity. Publication bias was assessed by the Egger’s test and funnel plots of the log OR versus the standard error were used as graphical representation.

To address possible small-study effect, funnel plots were visually inspected for asymmetry and the Egger’s test was used to assess publication bias, over and above any subjective evaluation, with *p* < 0.10 being considered statistically significant. In case of a significant publication bias, the Duval and Tweedie’s trim and fill method with the random-effect model was used to allow for the estimation of an adjusted effect size. In order to be as conservative as possible, the random-effect method was used to take into account both the within-study and between-study variability.

### 2.4. Subgroup Analyses and Meta-Regression Analyses

Separate analyses were performed by stratifying results for geographical area where each study was carried out. Analyses were repeated after excluding studies judged as low quality according to NOS score.

We performed meta-regression analyses in order to assess whether differences in the risk of all-cause mortality, CV mortality and CV events observed between highest and lowest white meat consumption categories were confounded by demographic (age, male gender) or clinical variables (body mass index, hypertension, diabetes mellitus, previous CV events, smoking habit) or follow-up duration. The regression models were carried out using differences in the risk of all-cause mortality, CV mortality and CV events observed between highest and lowest white meat consumption categories as the dependent variables (y) and the above-mentioned co-variates as independent variables (x). Comprehensive meta-analysis software (Version 3, Biostat, Englewood, NJ, USA, 2006) was used for the multivariate approach.

## 3. Results

### 3.1. Study Selection and Main Characteristics

**Figure 1:**
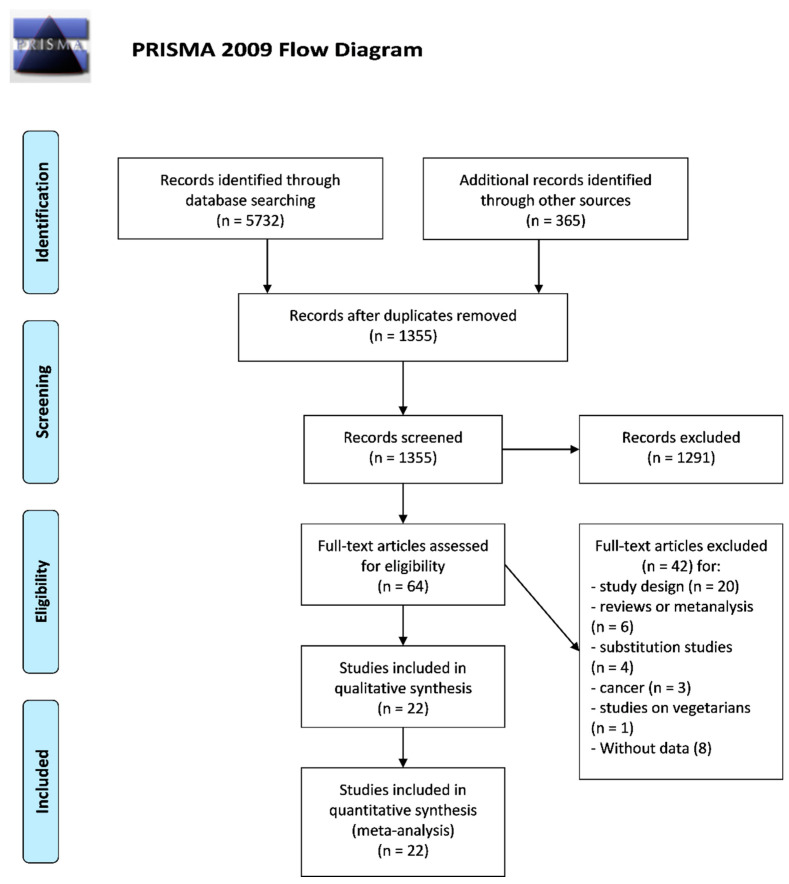
Preferred Reporting Items for Systematic reviews and Meta-Analyses (PRISMA) flow diagram.

After excluding duplicates, the search retrieved 1355 articles. Of these, 1291 were excluded because they were off the topic after scanning the title and/or the abstract; 42 because they were reviews/comments/case reports or lacked data of interest or did not match the inclusion and exclusion criteria. More in detail, among the studies excluded based on study design are also the study of Sinha et al. [11], because fish was combined with white meat, and the study of Sun et al. [34] which specifically focused on fried poultry only, at variance with all the other studies we included (Figure 1).

Among the included studies Lee et al. [10], Nagao et al. [35], Wurtz et al. [36], provided separate data for males and females; the study by Etemadi et al. [26] separately analyzed data for consumption of processed and unprocessed white meat and provided separate outcomes for ischemic heart disease and stroke; the study by Sluik et al. [27] provided separate data for diabetic and non-diabetic participants. In all these cases, the different populations were analyzed as separate datasets. Thus, a total of 22 studies [5,10,22,26,27,28,29,30,31,32,35,36,37,38,39,40,41,42,43,44,45,46] on 3,132,149 subjects were included in the final meta-analysis. The main characteristics of included studies are summarized in Table 1. Eight studies [22,26,31,37,38,39,44,45] were carried out in America, seven in Asia [10,28,29,32,35,41,46] and seven in Europe [5,27,30,36,40,42,43]. Eleven studies (14 data-sets) [10,26,27,31,39,40,41,42,43,44,46] reported data on all-cause mortality, 10 studies (15 datasets) [10,26,29,35,39,40,41,42,43,46] on CV mortality and 10 studies (11 datasets) [5,22,28,30,32,36,37,38,44,45] on non-fatal CV events. The evaluation of methodological quality of each study showed a median NOS score of 6.

### 3.2. All-Cause Mortality, CVD Mortality and CVD Events

**Figure 2:**
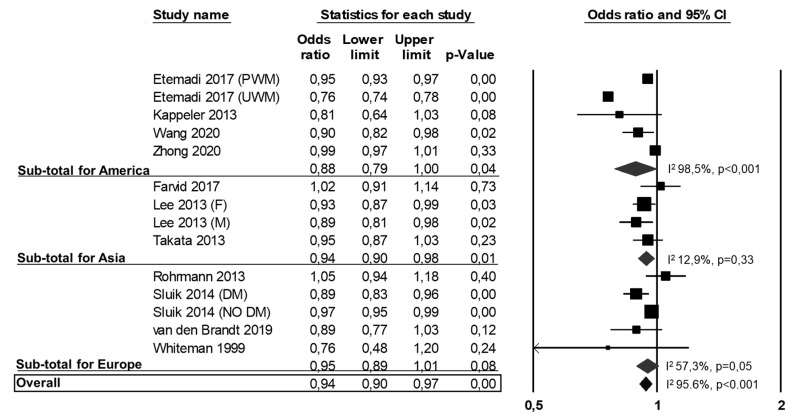
Association between white meat consumption (highest vs. lowest) and all-cause mortality. PWD: processed white meat; UWM: unprocessed white meat; F: females; M: males; DM: diabetes mellitus.

Of the 22 studies included in the meta-analysis, 11 studies reported data on all-cause mortality [10,26,27,31,39,40,41,42,43,44,46], four of these were conducted in America [12,26,39,44], three in Asia [10,41,46] and four in Europe [27,40,42,43]. One study reported separate data for processed and unprocessed white meat [26], one reported data by gender [10], and one reported data for people with or without diabetes [27]. Eleven studies were not included because there were no data on all-cause mortality [5,22,28,29,30,32,35,36,37,38,45]. The analysis of the 11 studies (14 datasets) on all-cause mortality showed a statistically significant lower mortality rate for subjects in the highest vs. lowest white meat consumption category (OR: 0.94, 95% CI: 0.90, 0.97, *p* < 0.001; Figure 2).

Heterogeneity among studies was significant (I^2^: 95.6%, *p* < 0.001) and it was not reduced by the exclusion of one study at a time. Results were consistently confirmed when specifically analyzing the 4 studies (5 datasets) from America [26,31,39,44] (OR: 0.88, 95% CI: 0.79, 1.00, *p* = 0.04; I^2^: 98.5%, *p* < 0.001; Figure 2) and the 3 studies (4 datasets) from Asia [10,41,46] (OR: 0.94, 95% CI: 0.90, 0.98, *p* = 0.01; I^2^: 12.9%, *p* = 0.33; Figure 2), while a non-significant association was found in the 4 studies (5 datasets) conducted in Europe [27,40,42,43] (OR: 0.95, 95% CI: 0.89, 1.01, *p* = 0.08; I^2^: 57.3%, *p* = 0.05; Figure 2).

**Figure 3:**
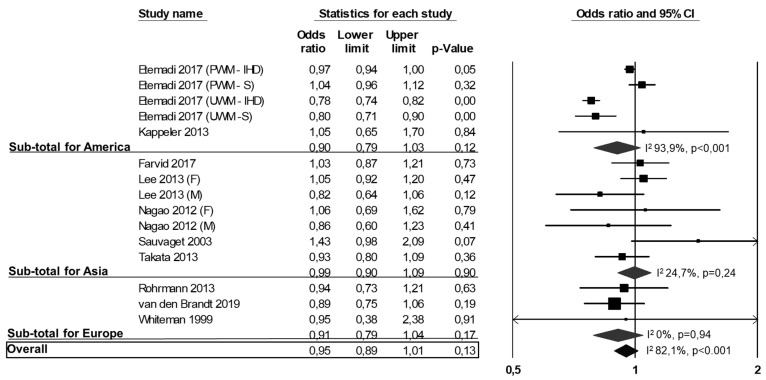
Association between white meat consumption (highest vs. lowest) and CVD mortality. PWD-IHD: processed white meat-ischemic heart disease; PWD-S: processed white meat-stroke; UWM-IHD: unprocessed white meat-ischemic heart disease; UWM-S: unprocessed white meat-stroke; F: females; M: males.

Of the 22 studies included in the meta-analysis, 10 reported data on CV mortality (i.e., stroke mortality, IHD mortality, or CVD mortality as a composite outcome) [10,26,29,35,39,40,41,42,43,46], two of these studies were conducted in America [26,39], five in Asia [10,29,35,41,46] and three in Europe [40,42,43]. One study reported data on processed and unprocessed white meat [26], and two studies reported separate data for males and females [10,35]. Twelve studies were not included because there were no data on CV mortality [5,12,22,27,28,30,32,36,37,38,44,45]. The analysis of the 10 studies (15 datasets) with CV outcomes showed no difference in CV mortality between subjects in the highest versus the lowest white meat consumption categories (OR: 0.95, 95% CI: 0.89, 1.01, *p* = 0.13; Figure 3).

Heterogeneity among studies was significant (I^2^: 82.1%, *p* < 0.001) and was not reduced by the exclusion of one study at a time. As shown in Figure 3, we found similar results when specifically analyzing studies from America [26,39] (OR: 0.90, 95% CI: 0.79, 1.03, *p* = 0.12; I^2^: 93.8%, *p* < 0.001), Asia [10,29,35,41,46] (OR: 0.99, 95% CI: 0.90, 1.09, *p* = 0.90; I^2^: 24.7%, *p* = 0.24) or Europe [40,42,43] (OR: 0.91, 95% CI: 0.79, 1.04, *p* = 0.17; I^2^: 0%, *p* = 0.94).

**Figure 4:**
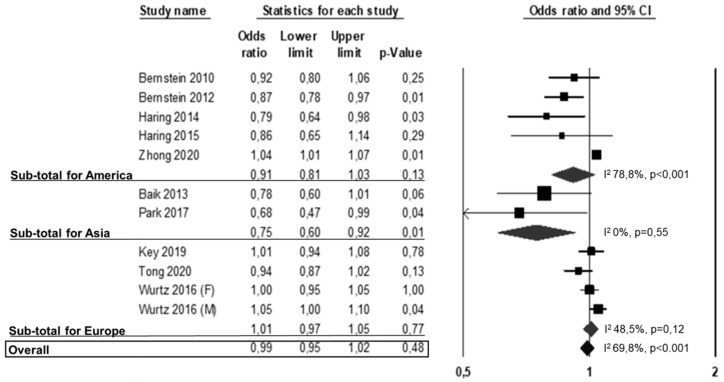
Association between white meat consumption (highest vs. lowest) and non-fatal CV events. F: females; M: males.

Of the 22 studies included in the meta-analysis, 10 studies (11 datasets) reported data on non-fatal CV events [5,22,28,30,32,36,37,38,44,45], five were from America [22,37,38,44,45], two from Asia [28,32] and three from Europe [5,30,36]. One study reported data on males and females [36]. Twelve studies were not included because there were no data on CV incidence [10,12,26,27,29,35,39,40,41,42,43,46]. On the overall no difference was observed in the risk of non-fatal CV events between subjects in the highest versus the lowest white meat consumption categories (OR: 0.99, 95% CI: 0.95, 1.02, *p* = 0.48; Figure 4).

Heterogeneity among studies was significant (I^2^: 69.8%, *p* < 0.001) and was not reduced by the exclusion of one study at a time. Results were confirmed when separately analyzing studies from America (OR: 0.91, 95% CI: 0.81, 1.03, *p* = 0.13; I^2^: 78.8%, *p* < 0.001) or Europe (Figure 4) (OR: 1.01, 95% CI: 0.97, 1.05, *p* = 0.77; I^2^: 48.5%, *p* = 0.12), while a significant risk reduction emerged from the Asian studies [28,32] (OR: 0.75, 95% CI: 0.60, 0.92, *p* = 0.01; I^2^: 0%, *p* = 0.55).

After excluding low quality studies (i.e., NOS < 6), the results were entirely confirmed for all-cause mortality (OR: 0.95, 95% CI: 0.91, 0.99, *p* = 0.02), CV mortality (OR: 0.94, 95% CI: 0.87, 1.02, *p* = 0.12) and non-fatal CV events (OR: 0.99, 95% CI: 0.96, 1.03, *p* = 0.78).

### 3.3. Publication Bias and Meta-Regressions

Funnel plot examination (Supplemental Figure S1) suggested the absence of publication bias and of small-study effect, confirmed by the Egger’s test for all-cause mortality and CVD mortality (Egger’s *p* = 0.713 and *p* = 0.852, respectively; Supplemental Figure S1). A significant publication bias (Egger’s *p* < 0.001) was observed for studies on CVD events. These results were confirmed by Duval and Tweedie’s trim and fill analysis.

Regression models showed that none of the clinical and demographic characteristics evaluated impacted on the difference in all-cause mortality between subjects in the highest versus lowest white meat consumption categories (Supplemental Table S2). No meta-regression analyses were performed for CV mortality and CV events due to the lack of statistically significant differences in the main analysis.

## 4. Discussion

Diet is one of the major modifiable factors that affect disease risk, thus it is of the greatest importance to identify dietary habits that decrease the risk of disease and death. The relationship of red and processed meat consumption with increased risk of all-cause death and incidence of CVD has been consistently demonstrated [5,10,12,13,14,15,17,31,37,38,39,41,42,46]; however, it remains unclear whether the adverse health effects associated with red and processed meat consumption are also shared by white meat [11,15,22,23]. We conducted a comprehensive meta-analysis of cohort studies exploring the relationship between withe meat consumption, total mortality and incident CV events (fatal or non-fatal).

The results have shown a 6% significantly lower all-cause mortality for subjects in the highest vs. the lowest white meat consumption category and an overall neutral association with CV mortality and morbidity. The findings for all cause death and CV death were fairly consistent when the analyses were stratified by geographical area (i.e., America, Asia, Europe) to partly account for different background diet and unmeasured lifestyle-related factors, including food preparation techniques. As for non-fatal CV events, the Asian studies indicated a significant risk reduction at variance with those from America and Europe. These should be further investigated due to the low number of meta-analyzed studies. There were not enough studies to perform the analyses by gender; however, the meta-regression analyses indicated that the clinical and demographic characteristics of the participants, including gender, did not impact on the difference in all-cause mortality.

In a previous meta-analysis of six studies, one of which also included fish in the white meat definition, Abete et al. have shown a weak and inverse association between white meat consumption and all-cause death in women only, and no relationship with CV mortality [15]. These conclusions, according to the authors themselves, were however weak, due to the small number and low quality of the meta-analyzed studies; furthermore, no data were available on non-fatal CV endpoints. Following the completion of the study by Abete et al. in 2014 several large prospective studies on the topic have been published, including some with non-fatal CV end points [5,26,28,30,36,38,42,44,46]. The present meta-analysis significantly expands current knowledge on the association of white meat consumption with total and CV mortality by including a much larger number of studies with a greater variety in geographical attribution, and studies with non-fatal cardiovascular outcomes. This allows a larger statistical power and a sub-analysis by geographical area and by type of outcome (i.e., fatal or non-fatal CVD). Furthermore, we better defined white meat by excluding studies on fish, due to the different health impact of fish and poultry consumption, which may have confounded prior analyses.

The interpretation of the effects of white meat consumption on health is a difficult task, as subjects consuming more white meat are, at the same time, consuming less red meat. On the other hand, people with a low red meat intake may prefer others protein sources, such as proteins from vegetable origin that could have per se a beneficial impact on cardiovascular health. However, it is important to underline that white meat, unlike products of vegetable origin, is a source of high-quality proteins and may therefore fully substitute red meat.

Furthermore, the background diet and the food preparation techniques, associated with the regular consumption of the different types of meat may also have a role which is difficult to account for. There are, however, plausible mechanisms which may partly mediate a different association of processed and red meat or white meat with health outcomes [47]. Meats are broadly classified into red (i.e., beef, pork, lamb) or white (i.e., chicken, turkey, rabbit) based on the contents of fat, cholesterol, and iron; furthermore, meats can be consumed fresh or processed with the addition of salt and chemicals. The different kinds of meats have important nutritional differences which may impact on health outcomes. Poultry meats, as compared with beef, lamb, or pork meat are characterized by a lower fat content, a more favorable fatty acid profile (i.e., mostly unsaturated fatty acids) [17,18,19] and a lower content of heme iron. Both saturated fat and heme iron are recognized factors involved in the promotion of atherosclerosis [48,49]. In addition, preservatives, such as sodium and nitrates, largely used in the preparation of processed meat, promote hypertension, insulin resistance and endothelial dysfunction, all of which are established CV risk factors.

Last, but not least, the study findings need to be evaluated also in the light of the growing awareness on the importance of the foods and beverages we produce, choose and consume in relation to their environmental sustainability [50,51]. It has been estimated that producing poultry meat has a substantially lower ecological impact than producing beef meat [50,51]. Therefore, promoting a moderate consumption of unprocessed white meat, particularly if in substitution of red and processed meat, emerges as a potential strategy to improve human health and, at the same time, limit environmental deterioration.

This study has some strengths. First, it includes a large number of studies and participants (11 studies for all cause death, 10 studies for CV death, and 10 for non-fatal CV events), thus allowing sufficient statistical power, second it provides data on non-fatal CV events which were not available before, furthermore, the analysis by geographical area confers internal consistency to the results by partly adjusting for unmeasured cultural and lifestyle related factors including background diet and procedures of food preparation.

The potential limitations need also to be discussed. First, we must acknowledge that studies included in this meta-analysis have different inclusion and exclusion criteria and therefore subjects with different clinical and demographic characteristics were considered in the overall analysis. Moreover, meta-analyses are performed on aggregate data and the multivariate approach allows for the adjustment for some, but not all, potential confounders. Thus, although meta-regression models were used to refine our analyses by assessing the influence of most clinical and demographic variables on the observed results, caution is necessary in the interpretation of results due to potential residual confounding. Finally, our results were affected by a significant heterogeneity. Although it was not possible to definitively establish sources of such heterogeneity, all findings were substantially confirmed by appropriate sensitivity and subgroup analyses and the impact of clinical and demographic variables on the study results was evaluated by means of meta-regression models. Furthermore, we excluded the presence of publication bias by using different methods, and in case of significant bias our results were confirmed after trimming and imputing studies. In addition, we found heterogeneity among studies in the definition of white meat intake in the lowest and the highest consumption group (for example, servings per day or per week, or grams per day) and in some studies there was missing information regarding type of white meat (for example, total white meat, or only chicken). Moreover, the potential confounding of cooking methods and preparation techniques could not be assessed.

## 5. Conclusions

In conclusion, the study shows a robust and inverse association between the consumption of unprocessed white meat and all-cause mortality, and a neutral association with CV mortality and morbidity. These findings highlight the importance of differentiating the meat types and suggest that white meat might be a ‘healthy’ and environmentally more sustainable alternative to red and processed meat consumption. Most people consume meats on a regular basis and therefore, notwithstanding the relatively modest risk reduction (6%), the findings of this study have relevant public health implications and provide evidence to inform dietary guidelines for a healthy and environmentally sustainable nutrition.

## Figures and Tables

**Table 1:** Characteristics of the prospective cohort studies included in the analysis

Author, Publication Year, Location	Participants	Dietary Intake Assessment Method	Total Cases	Highest vs. Lowest Intake	Outcome	HR for the Highest vs. Lowest Intake	Adjusted Variables
Baik, 2013, Asia	n 9026 (M 4694, F 4332) Age 52 years Follow-up 8 years *	FFQ (103 items)	CVD: 352	Predefined categories ≥ 1 serving/week vs. 0	Incident CVD	0.78 (95% CI 0.60, 1.01)	sex, age, systolic blood pressure, antihypertensive treatment, total cholesterol in serum, HDL cholesterol in serum, smoking status, diabetes mellitus, BMI, legumes intake, carbonated soft drink intake and green tea intake
Bernstein, 2010, America	n 84136 (F) Age 58 years Follow-up 26 years	FFQ	IHD: 3162	0.56 serving/day vs. 0.07 serving/day ^1^	Incident IHD	0.92 (95% CI 0.8, 1.06)	age, time period, total energy, cereal fiber, alcohol, trans fat, BMI, cigarette smoking, menopausal status, parental history of early myocardial infarction, multivitamin use, vitamin E supplement use, aspirin use at least once per week, physical exercise
Bernstein, 2012, America	n 127160 (M 43150, F 84010) Age 59 years Follow-up 24.6 years	FFQ (61–131 items)	Stroke: 4030 Ischemic stroke: 2212 Hemorrhagic stroke: 693	0.72 serving/day vs. 0.14 serving/day (M) ^2^ 0.54 serving/day vs. 0.14 serving/day (F) ^3^	Incident Stroke Incident Ischemic stroke Incident Hemorrhagic stroke	0.87 (95% CI 0.78, 0.97) 0.89 (95% CI 0.76, 1.03) 0.74 (95% CI 0.52, 1.06)	BMI, cigarette smoking, physical exercise, parental history of early myocardial infartcion (<60 y), menopausal status (only women), multivitamine use, vitamin E supplement use, aspirin use at least once per wk, total energy intake, cereal fiber, alcohol, transfat, fruit and vegetables and other protein sources
Etemadi, 2017, America	n 536 969 (M 316505, F 220464) Age 62 years Follow-up 15.6 years *	FFQ (124 items)	All-cause mortality: 128524 IHD mortality: 34723 Stroke mortality: 5837		All-cause mortality IHD mortality Stroke mortality	0.95 (95% CI 0.93, 0.96) (PWM) 0.76 (95% CI 0.74, 0.78) (UWM) 0.97 (95% CI 0.94, 1.00) (PWM) 0.78 (95% CI 0.74, 0.81) (UWM) 1.04 (95% CI 0.96, 1.12) (PWM) 0.8 (95% CI 0.71, 0.89) (UWM)	sex, age, marital status, ethnicity, education, fifths of composite deprivation index, perceived health at baseline, history of heart disease, stroke, diabetes, cancer, smoking status, BMI, vigorous physical activity, usual activity throughout day, alcohol consumption, fruit and vegetable intakes, total energy intake and total meat intake
Farvid, 2017, Asia	n 42 403 (M 18318, F 24085) Age 51.6 years Follow-up 8.1 years *	FFQ (116 items)	All-cause mortality: 3291 CVD mortality: 1467 IHD mortality: 764 Stroke mortality: 507	1.33 serving/day vs. 0.11 serving/day ^4^	All-cause mortality CVD mortality IHD mortality Stroke mortality	1.02 (95% CI 0.91, 1.14) 1.03 (95% CI 0.87, 1.21) 0.97 (95% CI 0.77, 1.22) 1.06 (95% CI 0.8, 1.39)	age, ethnicity, education, marital status, residency, smoking status, opium use, alcohol, BMI, systolic blood pressure, occupational physical activity, family history of cancer, wealth score, medication and energy intake
Haring, 2014, America	n 12 066 (M 5333, F 6733) Age 53.8 years Follow-up 22 years *	FFQ (66 items)	IHD: 1147	0.8 serving/day vs. 0.1 serving/day	Incident IHD	0.79 (95% CI 0.64, 0.98)	age, sex, race, study center, total energy intake, smoking, education, systolic blood pressure, use of antihypertensive medicatione, HDLcholesterol, total cholesterol, use of lipid lowering medication, BMI, waist to hip ratio, alcohol intake, sports related physical activity, leisure related physical activity, CHO intake, fiber intake and magnesium intake
Haring, 2015, America	n 11 601 (M 5116, F 6485) Age 53.8 years Follow-up 22.7 years *	FFQ (66 items)	Stroke: 699 Ischemic stroke: 598 Hemorrhagic stroke: 114	0.8 serving/day vs. 0.07 serving/day	Incident Stroke Incident Ischemic stroke Incident Hemorrhagic stroke	0.86 (95% CI 0.65, 1.14) 0.94 (95% CI 0.7, 1.27) 0.56 (95% CI 0.26, 1.2)	age, sex, race, study center, total energy intake, cigarette years, education, systolic blood pressure, use of antihypertensive medicatione, HDLcholesterol, total cholesterol, use of lipid lowering medication, BMI, waist to hip ratio, alcohol intake, sports related physical activity, leisure related physical activity, CHO intake, fiber intake and magnesium intake
Kappeler, 2013, America	n 17 611 (M 8239, F 9372) Age 41 years Follow-up 22 years	FFQ (81 items)	All- cause mortality: 3683 CVD mortality: 1554	≥13 times/months vs. 0	All- cause mortality CVD mortality	0.81 (95% CI 0.64, 1.03) 1.05 (95% CI 0.65, 1.71)	age, race, sex, smoking status, alcohol consumption, physical activity, socioeconomic status, BMI, marital status, fruit and vegetable intake, history of hypertension, diabetes, hypercolesterolemia, use of aspririn and ibuprofen, use of mineral and vitamin supplements, family history of diabetes, or hypercholesterolemia and hormone replacement therapy and oral contraceptive use (only women)
Key, 2019, Europe	n 409 885 (M 106751, F 303134) Age 51.7 years Follow-up 12.6 years	FFQ (EPIC study)	IHD: 7198	46 g/die vs. 0 g/die	Incident IHD	1.01 (95% CI 0.94, 1.1)	age, smoking status and number of cigarettes-day, diabetes mellitus, hypertension, hyperlipidemia, physical activity level, employment status, educational level, BMI, alcohol intake, energy intake, fruit and vegetable intake, sugars intake and fiber from cereals intake
Lee, 2013, Asia	n 296 721 (M 112310, F 184411) Age n.a. Follow-up 6.6–15.6 years	FFQ (6–17 items)	All- cause mortality: 14326 (M) and 9957 (F) CVD mortality: 3579 (M) and 2794 (F)	Mean intake: 4.6–22.3 g/day (M) 2.8–15.4 g/day (F)	All-cause mortality CVD mortality	0.89 (95% CI 0.81, 0.98) (M) 0.93 (95% CI 0.86, 0.99) (F) 0.82 (95% CI 0.64, 1.06) (M) 1.05 (95% CI 0.92, 1.18) (F)	age, BMI, education level, smoking status, rural/urban residence, alcohol intake, fruit and vegetable intake and total energy intake
Nagao, 2012, Asia	n 51 638 (M 20466, F 31217) Age 55.7 (M) and 56.1 (F) years Follow-up 18.4 years *	FFQ (40 items)	IHD mortality: 301 (M) and 236 (F)	27.3 g/day vs.1.9 g/day (M) 22.4 g/day vs. 1.5 g/day (F)	IHD mortality	0.86 (95% CI 0.6, 1.23) (M) 1.06 (95% CI 0.69, 1.62) (F)	age, BMI, ethanol intake, perceived mental stress, walking time, sports participation time, education years, history of hypertension and diabetes, total energy and energy-adjusted food (rice, fish, soy, vegetables and fruits) intakes
Park, 2017, Asia	n 9311(M 4461, F 4850) Age 52.1 years Follow-up 7.8 years *	FFQ (110 items)	CVD: 486	1.41 serving/week vs. 0	Incident CVD	0.68 (95% CI 0.47, 0.99)	age, sex, educational level, household income, residential area, smoking status, alcohol intake, BMI, physical activity, total energy intake and total fruit and vegetable intake
Rohrmann, 2013, Europe	n 448 568 (M 127321, F 321247) Age 51.3 years Follow-up 12.7 years	FFQ (EPIC study)	All-cause mortality:26344 CVD mortality: 5556	50.3 g/day vs. 9.7 g/day (M) 35.6 g/day vs.10.5 g/day (F)	All-cause mortality CVD mortality	1.05 (95% CI 0.94, 1.18) 0.94 (95% CI 0.73, 1.21)	education, body weight, body height, total energy intake, alcohol consumption, physical activity, smoking status, smoking duration and other meat intake
Sauvaget, 2003, Asia	n 32049 Age 56 years * Follow-up 16 years	FFQ (22 items)	Stroke mortality: 1462	17.9 ± 39.61 g/day vs. 4.72 ± 24 g/day	Stroke mortality	1.43 (95% CI 0.98, 2.1)	city, radiation dose, self-reported BMI, smoking status, alcohol habits, education level, history of diabetes or hypertension
Sluik, 2014, Europe	n 265 295 (M 107011, F 158284) Age 57.4 (with DM) and 51.8 (w/o DM) Follow-up 9.9 years *	FFQ (300–500 items)	All-cause mortality: 830 (with DM) and 12135 (w/o DM)	10 g/day vs. 0	All-cause mortality	0.89 (95% CI 0.83, 0.96) (with DM) 0.97 (95%CI 0.95, 1.00) (w/o DM)	sex, prevalence of heart disease, cancer or stroke, educational attainment, diabetes medication use (only for DM) and the following when there were no exposure variables (alcohol consumption, smoking behaviour, physical activity and underlying dietary patterns)
Takata, 2013, Asia	n 134 290 (M 61128, F 73162) Age 55.5 (M) and 52.9 (F) years Follow-up 8.6 years *	Gender specific FFQ (81 items for M and 77 items for F)	All- cause mortality: 6943 CVD mortality: 2163 IHD mortality: 590 Ischemic stroke mortality: 504 Hemorrhagic stroke mortality: 530	37.9 g/day vs. 0.9 g/day (M) 33.8 g/day vs. 1.4 g/day (F)	All-cause mortality CVD mortality IHD mortality Ischemic stroke mortality Hemorrhagic stroke mortality	0.95 (95% CI 0.87, 1.03) 0.93 (95% CI 0.79, 1.08) 1.08 (95% CI 0.81, 1.44) 0.99 (95%CI 0.72, 1.37) 1.05 (95%CI 0.77, 1.42)	age, total energy intake, income, occupation, education level, comorbidity index, physical activity level, total vegetable intake, total fruit intake, fish intake, red meat intake, smoking history and alcohol consumption (only men)
Tong, 2020, Europe	n 418 329 (M 140117, F 278212) Age 50.9 years Follow-up 12.7 years	FFQ (EPIC study)	Stroke: 7378 Ischemic stroke: 4281 Hemorrhagic stroke: 1430	44.6 g/day vs. 0 *	Stroke Ischemic stroke Hemorrhagic stroke	0.94 (95%CI 0.87, 1.02) 0.97 (95%CI 0.88, 1.07) 0.97 (95%CI 0.82, 1.16)	age, smoking status and number of cigarettes per day, history of diabetes, prior hypertension, prior hyperlipidaemia, Cambridge physical activity index, employment status, level of education completed, current alcohol consumption, BMI, and observed intake of energy, and stratified by sex and EPIC centre.
van den Brandt, 2019, Europe	n 120 852 Age 61.4 years Follow-up 10 years	FFQ	All-cause mortality: 8823 CVD mortality: 2985	22.8 g/day vs. 0	All-cause mortality CVD mortality	0.89 (95% CI 0.77, 1.03) 0.89 (95%CI 0.75, 1.06)	age, sex, cigarette smoking status, number of cigarettes smoked per day, years of smoking, diabetes, body height, BMI, non-occupational physical activity, highest level of education, intake of alcohol, vegetable and fruit, energy, use of nutritional supplements and postmenopausal HRT (only women)
Wang, 2020, America	n 9286 (M) Age 72.1 years Follow-up 23 years	FFQ (68 items)	All-cause mortality: 4682	3.5 serving/week * vs. 0.6 serving/week *	All-cause mortality	0.9 (95% CI 0.82, 0.98)	age, calendar year of prostate cancer diagnosis, tumor extent, Gleason score, nodal involvement, education, family history of prostate cancer, history of PSA testing, BMI, smoking status, physical activity, history of diabetes, CVD history and other cancer, total fruit and vegetable intake, energy intake, egg intake, fish intake, processed and unprocessed meat intake and red meat intake.
Whiteman, 1999, UK	n 10 055 Age n.a. Follow-up 9 years	FFQ	All-cause mortality: 472 IHD mortality: 96	4–7 days/week vs. <1 day/week	All-cause mortality IHD mortality	0.76 (95% CI 0.48, 1.19) 0.95 (95% CI 0.38, 2.38)	gender, smoking and age group
Wurtz, 2016, Europe	n 55 171 (M 26029, F 29142) Age 55 (M) * and 56 (F) * Follow-up 13.5 (M) * and 13.6 (F) *	FFQ (192 items)	IHD: 1694 (M) and 656 (F)	21.4 g/day vs. 0	Incident IHD	1.05 (95% CI 1.00, 1.11) (M) 1.0 (95% CI 0.9, 1.1) (F)	age, total energy intake, alcohol abstinence, alcohol intake, BMI, waist circumference, smoking status and amount, physical activity, duration of schooling, menopausal status, use of hormone replacement therapy (only women), investigated food items, fruits, sweets, soft drinks, lean dairy products, fatty dairy products, potato chips, refined cereals, wholegrain cereals, nuts
Zhong, 2020, America	n 29 682 (M 13168 F 16514) Age 53.7 years Follow-up 19 years *	FFQ	All-cause mortality: 8875 CVD: 6963	0.29 serving/day vs. 0 ^5^	All-cause mortality Incident CVD	0.99 (95% CI 0.97, 1.02) 1.04 (95% CI 1.01, 1.06)	age, sex, race/ethnicity, educational level, total energy, smoking status, smoking pack-years, cohort-specific physical activity z score, alcohol intake, hormone therapy, fruits, legumes, potatoes, other vegetables, excluding legumes and potatoes, nuts and seeds, whole grains, refined grains, low-fat dairy products, high-fat dairy products, sugar-sweetened beverages, eggs, and 3 of the 4 food types (processed meat, unprocessed red meat, poultry, and fish

CVD: cardiovascular diseases; IHD: ischemic heart diseases; FFQ: food frequencies questionnaires; BMI: body mass index; PWM: processed white meat; UWM: unprocessed white meat; DM: diabetes mellitus; M: male; F: female; * Median value. 1. Serving: chicken w or w/o skin = 8–3 oz; chicken or turkey dog = 1 chicken or turkey dog; chicken liver = 1 oz. 2. Serving size for males: chicken or turkey w or w/o skin= 6–3 oz; chicken sandwich = not quantified; chicken or turkey dog = 1 chicken or turkey dog. 3. Serving size for females: chicken w or w/o skin = 6–8 oz; chicken or turkey w or w/o skin = 6–3 oz; chicken or turkey dog = 1 chicken or turkey dog. 4. Serving size: 85 g of cooked chicken. 5. Serving size: 4 oz.

## Supplementary Materials

The following are available online at https://www.mdpi.com/2072-6643/13/2/676/s1, Figure S1: Funnel plots of the log OR versus the standard error for studies evaluating all-cause mortality (Panel A), CVD mortality (Panel B), and CVD events (Panel C), Table S1: Quality of studies assessment (Newcastle-Ottawa scale) for included studies, Table S2: Meta-regression analyses for all-cause mortality.
