# Supplementary material for: White Meat Consumption, All-Cause Mortality, and Cardiovascular Events: A Meta-Analysis of Prospective Cohort Studies
Source: Nutrients. 2021 Feb 20;13(2):676. doi: 10.3390/nu13020676 (PMC7924043; doi:10.3390/nu13020676)
Supplement: Supplementary file 1 [file nutrients-13-00676-s001.pdf]

**Supplemental Table 1.** Quality of studies assessment (Newcastle-Ottawa scale) for included studies

| | Representativeness of the exposed cohort | Selection of the non exposed cohort | Ascertainment of exposure | Demonstration that outcome of interest was not present at start of study | Comparability of cohorts | Assessment of outcome | Length of follow-up | Lost at follow up | Total |
|---|---|---|---|---|---|---|---|---|---|
| Baik 2013 | ✸ | ✸ | | ✸ | ✸ | | ✸ | | 5/8 |
| Bernstein 2010 | | ✸ | | ✸ | ✸ | ✸ | ✸ | ✸ | 6/8 |
| Bernstein 2012 | ✸ | ✸ | | ✸ | ✸ | ✸ | ✸ | ✸ | 7/8 |
| Etemadi 2017 | ✸ | ✸ | | ✸ | ✸ | ✸ | ✸ | | 6/8 |
| Farvid 2017 | ✸ | ✸ | | ✸ | ✸ | ✸ | ✸ | ✸ | 7/8 |
| Haring 2014 | ✸ | ✸ | | ✸ | ✸ | | ✸ | | 5/8 |
| Haring 2015 | ✸ | ✸ | | ✸ | ✸ | ✸ | ✸ | | 6/8 |
| Kappeler 2013 | ✸ | ✸ | | ✸ | ✸ | ✸ | ✸ | | 6/8 |
| Key 2019 | ✸ | ✸ | | ✸ | ✸ | ✸ | ✸ | ✸ | 7/8 |
| Lee 2013 | | ✸ | | ✸ | ✸ | | | | 3/8 |
| Nagao 2012 | ✸ | ✸ | | ✸ | ✸ | ✸ | ✸ | | 6/8 |
| Park 2017 | ✸ | ✸ | | ✸ | ✸ | ✸ | ✸ | | 6/8 |
| Rohrmann 2013 | ✸ | ✸ | | ✸ | ✸ | ✸ | ✸ | ✸ | 7/8 |
| Sauvaget 2012 | ✸ | ✸ | | ✸ | ✸ | ✸ | ✸ | | 6/8 |
| Sluik 2017 | ✸ | ✸ | | ✸ | ✸ | ✸ | ✸ | | 6/8 |
| Takata 2013 | ✸ | ✸ | | ✸ | ✸ | ✸ | | ✸ | 6/8 |
| Tong 2020 | ✸ | ✸ | | ✸ | ✸ | ✸ | ✸ | | 6/8 |
| Van den Brandt 2019 | ✸ | ✸ | | ✸ | ✸ | ✸ | ✸ | | 6/8 |
| Wang 2020 | | ✸ | | ✸ | ✸ | ✸ | ✸ | ✸ | 6/8 |
| Whiteman 1999 | ✸ | ✸ | | ✸ | ✸ | ✸ | ✸ | ✸ | 7/8 |
| Wurtz 2016 | ✸ | ✸ | | ✸ | ✸ | ✸ | ✸ | ✸ | 7/8 |
| Zhong 2020 | ✸ | ✸ | | ✸ | ✸ | ✸ | ✸ | ✸ | 7/8 |

**Supplemental Figure 1.** Funnel plots of the log OR versus the standard error for studies evaluating all-cause mortality (Panel A), CVD mortality (Panel B), and CVD events (Panel C).

A

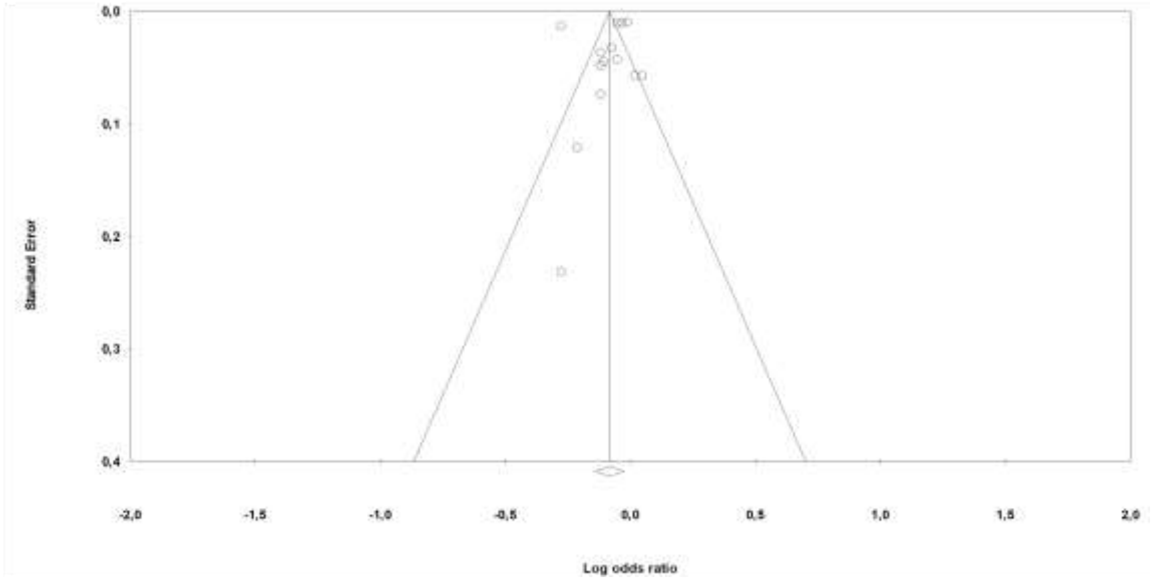


B

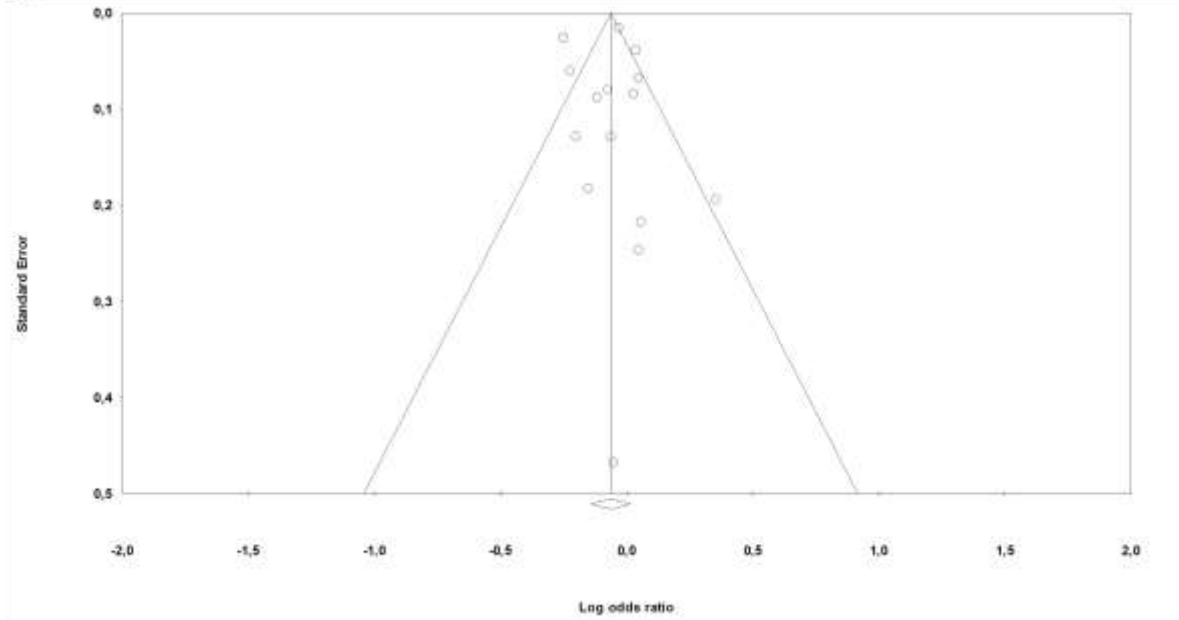

C

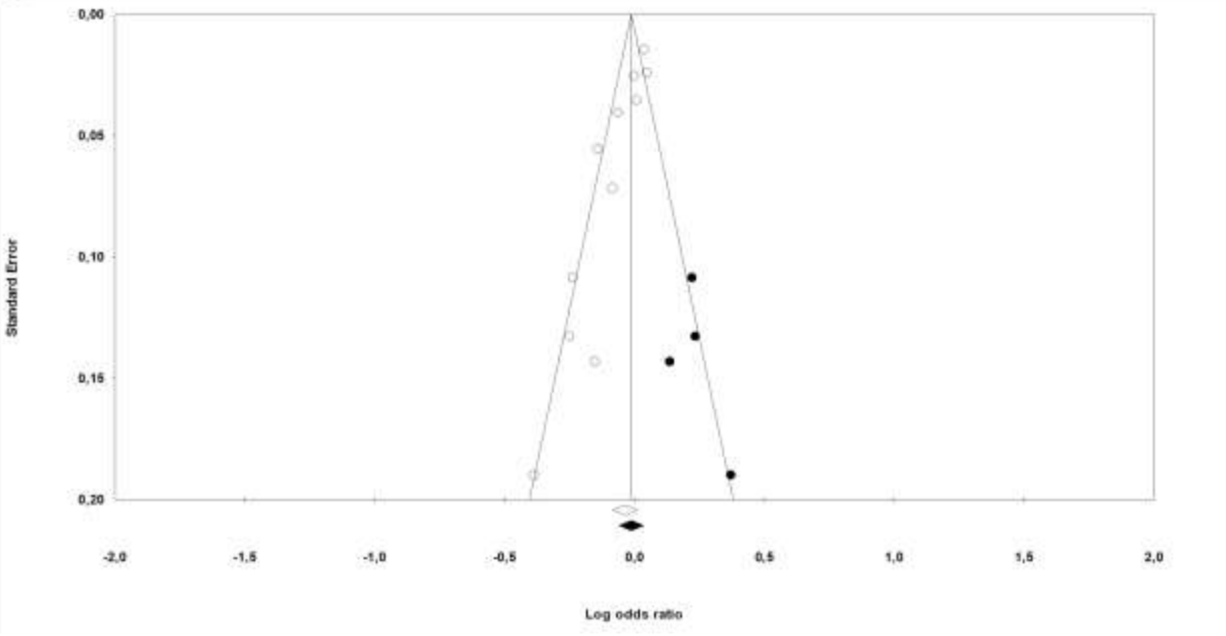
0,00
0,05
0,10
0,15
0,20
Standard Error
-2,0
-1,5
-1,0
-0,5
0,0
0,5
1,0
1,5
2,0
Log odds ratio

**Supplemental Table 2.** Metaregression analyses for all-cause mortality.

| | **All-cause mortality** | |
|---|---|---|
| | Z-value | P-value |
| Age | -0,92 | 0,358 |
| Male gender | -0,97 | 0,334 |
| BMI | -1,10 | 0,272 |
| Hypertension | -1,83 | 0,067 |
| Diabetes | -0,34 | 0,736 |
| CAD | -0,91 | 0,363 |
| Smoking | 0,71 | 0,475 |
| Follow-up | -0,62 | 0,534 |
